# HDAC2 restrains IGF2BP1-associated m6A dysregulation in cervical cancer immunotherapy resistance

**DOI:** 10.1371/journal.pone.0355606

**Published:** 2026-08-27

**Authors:** Qiuhong Deng, Zhi Wang, Jine Zhao, Ning Li, Linghua Zhang, Wanxia He, Yongmei Zhang

**Affiliations:** Department of Gynecology, The Second Clinical Hospital of Ningxia Medical University, The First People’s Hospital of Yinchuan, Ningxia, China; Huazhong Agriculture University, CHINA

## Abstract

**Background:**

Cervical cancer (CC) immunotherapy resistance remains a critical clinical challenge. Although immune checkpoint blockade shows efficacy in subsets of patients, the epigenetic and post-transcriptional mechanisms driving resistance are poorly understood. Insulin-like growth factor 2 mRNA-binding protein 1 (IGF2BP1), an m6A “reader,” is implicated in tumor progression, but its role in CC immunotherapy resistance is undefined. The aim of this study was to elucidate the function of epigenetic regulator IGF2BP1 and HDAC2 in CC acquired immunotherapy resistance.

**Methods:**

Multi-omics analysis of GEO/TCGA datasets identified immunotherapy response related genes. Functional validation employed siRNA knockdown, overexpression, in vitro co-culture assay treated with anti-PD-1. Molecular mechanisms were assessed via RIP/MeRIP-qPCR, ChIP-qPCR, and luciferase reporter experiments.

**Results:**

IGF2BP1 was significantly upregulated in CC tissues and cell lines (HeLa, SiHa, CaSki, C-33A), correlating with poor survival (P < 0.001). IGF2BP1 knockdown suppressed proliferation and sensitized tumors to anti-PD-1 via enhancing CD8 ⁺ T cell infiltration and IFNγ secretion. Mechanistically, IGF2BP1 stabilized immune-response genes and stemness program genes (PD-L1, WNT4, POU5F1, MYC) via m6A-dependent mRNA processing. HDAC2 was differentially associated with PD-1 treatment response, suggesting a context-dependent function and potential value as an exploratory biomarker of immunotherapy responsiveness. HDAC2 repressed IGF2BP1 transcription by erasing H3K27ac marks at its promoter. Targeting above axis synergistically enhanced T cells activation and toxic function.

**Conclusions:**

We unveil an HDAC2/IGF2BP1/m6A axis is associated with cervical cancer immunotherapy resistance. HDAC2 loss derepresses IGF2BP1, stabilizing immunosuppressive and stemness-related transcripts via m6A modification. Targeting this axis partially restores PD-1 efficacy, providing a novel insight of cervical cancer immunotherapy resistance.

## Introduction

Cervical cancer (CC) remains a significant global health burden, accounting for substantial cancer-related mortality among women despite advances in screening and vaccination programs [1–3]. While immune checkpoint inhibitors (ICBs) targeting the PD-1/PD-L1 axis have demonstrated efficacy in multiple malignancies, their clinical benefit in CC is limited by primary and acquired resistance mechanisms [4–6]. Emerging evidence implicates dysregulated epigenetic and post-transcriptional networks remodel immunosuppressive tumor microenvironments [7–10], yet the precise molecular determinants of immunotherapy resistance in CC remain incompletely defined. N6-methyladenosine (m6A) is the most abundant internal mRNA modification in mammalian cells and has been closely linked to leukemogenesis [11,12]. Dysregulation of the m6A machinery has increasingly been implicated not only in tumor initiation and progression but also in resistance to anticancer treatment [13,14]. Particularly, aberrant m6A regulation can alter tumor-cell sensitivity to chemotherapy and IFN-γ-mediated killing [15]. m6A reader YTHDF1 play crucial role in immune evasion and ICB resistance through MHC-I degradation promoting [16]. However, the function of m6A key machinery protein in cervical cancer immunotherapy resistance still unclear. The RNA-binding protein IGF2BP1, also a well-established m6A “reader,” can recognize m6A modifications in mRNAs and determine RNA fate by regulating mRNA processing, translation and stability [14,17]. Intriguingly, our bioinformatic analysis of immunotherapy-resistant CC cohorts revealed IGF2BP1 as a top-ranked resistance-associated gene, with elevated expression correlating significantly with poor patient survival. Paradoxically, while histone deacetylase HDAC2 typically functions as an oncogene in solid tumors, our multi-cohort interrogation unexpectedly identified HDAC2 as a potent immunotherapy-sensitizing factor enriched in ICB-responsive CC samples, suggesting context-dependent tumor-suppressive functions.

HDAC2 is a class I histone deacetylase that regulates chromatin accessibility and transcription through the removal of acetyl groups from histone and non-histone proteins [18]. Although HDAC2 is frequently associated with tumor-promoting activity, its function is context dependent. La Noce et al. reported that HDAC2 depletion promoted osteosarcoma stemness and cancer stem-cell phenotypes in vitro and in vivo [19]. Here, we elucidate a novel HDAC2/IGF2BP1 regulatory axis is associated with CC progression and anti-PD-1 resistance. We demonstrate that constitutive IGF2BP1 activation drives tumor proliferation and immune evasion by stabilizing m6A-modified transcripts of key immunosuppressive (e.g., *PD-L1*, *CXCR4*) and stemness-related genes (e.g., *WNT4*, *POU5F1*, *MYC*) in an METTL3/14-dependent manner. Mechanistically, HDAC2 directly represses IGF2BP1 transcription by catalyzing H3K27 deacetylation at its promoter, thereby attenuating downstream oncogenic programs. Critically, targeting this axis synergistically enhances T cell activation and achieves improved toxicity in vitro co-culture system. Our study provides a rationale for further investigation.

## Method

### Cell lines

Human cervical cancer cell lines HeLa, C-33A and SiHa and the normal human cervical epithelial cell line HcerEpic were obtained from the National Collection of Authenticated Cell Cultures, and CaSki cells were purchased from ATCC. Cell-line identity was confirmed by short tandem repeat (STR) and all cell lines were confirmed to be free of mycoplasma contamination using Mycoplasma PCR Detection Kit (Beyotime, China). These cells were cultured in DMEM high-glucose medium with the addition of 10% fetal bovine serum (Thermo Fisher Scientific, USA) and 100 U/mL amphotericin B-streptomycin–penicillin solution (Thermo Fisher Scientific, USA). The cells cultured in 37℃ incubator containing 5% CO_2_.

### Cell transfection

Briefly, siRNA (RiboBio, Guangzhou, China) diluted with Opti-MEM and transfected with Lipo-fectamine 3000 (Invitrogen, USA) when the cell confluency was 60%−80%. Cells incubated 48h for harvested RNA or following experiment. For overexpression, vector plasmid or HDAC2 expression constructs were diluted in Opti-MEM and transfected using Lipo-fectamine 2000 (Invitrogen, USA) when the cell confluency reached 80%. CCK8 and colony formation assays performed after transfection 24 h.

### Lentiviral generation and infection

Lentiviral particles of sh-*NC* and sh-*IGF2BP1* for humans were purchased from Santa Cruz Biotechnology. pLKO.1 plasmids of sh-*NC*, sh-*IGF2BP1* were obtained from Sigma. Lentivirus was produced by co-transfection into HEK-293T cells with lentiviral vectors in combination with the pCMVdelta8.2 packaging vector and pVSV-G envelope vector using Lipo-fectamine 3000 (Invitrogen, USA). Virus-containing supernatants were collected 24–48 h after transfection and used to infect recipients. Target cells were infected in the presence of Polybrene (8 μg/ml) (Sigma-Aldrich) and selected with puromycin (Santa Cruz Biotechnology, 1 μg/ml) for 6 days [20].

### Compounds

ITSA-1 (HY-100508) were used to treat cervical cell lines. CCK8 and IC50 assay evaluating the toxicity of ITSA-1 on cervical cancer cells.

### Real-time quantitative PCR analysis (RT-qPCR)

Total RNA was extracted from cultured cells using TRIzol reagent (Invitrogen, USA) according to the manufacturer’s instructions. RNA concentration was measured using the NanoDrop 2000 (USA). Subsequently, total RNA was reverse transcribed into cDNA using the PrimeScript™ RT Reagent Kit (Takara, Japan). RT-qPCR was performed on Roche LightCycler 480II Real-Time PCR Detection System (Roche, Basel, Switzerland). Gene expression level was calculated using the 2 ^− ΔΔCt^ method normalized to GAPDH levels.

### Ribonucleoprotein-immunoprecipitation assay (RIP)

Cells were homogenized in RIP lysis buffer (20mM Tris-HCl pH7.4, 150mM NaCl, 1% (v/v) IGEPAL® CA-630 (Millipore Sigma, Cat# I8896), 1mM EDTA, 0.5 mM DTT, cOmplete^TM^ EDTA-free Protease Inhibitor Cocktail Tablets (Merck, Cat# 4693132001) and RNase inhibitor (Beyotime, R0102), and spun at 14,000 rpm for 10 minutes to remove the debris. 50 μL of empty Dynabeads® Protein G was washed by Citrate-Phosphate Buffer and then incubated with 5ug anti-IGF2BP1 (Proteintech, AP22803−1) antibody for 4 hours at 4°C with rotation. The prewashed Dynabeads Protein G/A-Ig complex was incubated with the precleared lysates at 4°C overnight with rotation. The beads were then washed with RIP wash buffer twice and RIP high-salt wash buffer twice and then the RNA was eluted using 1 mL of TRIzol solution.

### CHIP-qPCR

The SimpleChIP® Plus Sonication Chromatin IP Kit (Cell Signaling Technologies, Cat No. #56383) was exploited to conduct the ChIP assay as previously [21]. HeLa or C-33A cells (1  ×  10^7^) were fixed with 1% formaldehyde for 10 min at room temperature, and the reaction was quenched by adding glycine to a final concentration of 125 mM for 5 min. Cells were washed twice with ice-cold PBS and collected by centrifugation. After sonication, the lysates were clarified by centrifugation at 21,000 × g for 10 min at 4°C. An aliquot of chromatin was reverse-crosslinked and analyzed by agarose gel electrophoresis to confirm that the majority of DNA fragments ranged from approximately 200–1000 bp. The lysate incubated overnight at 4°C with rotation with anti-HDAC2 antibody (Cell Signaling Technologies, #2540, 1:200), anti-H3K27ac antibody (Cell Signaling Technology, 8173;), or normal rabbit IgG (Proteintech, 30000–0-AP, 1:100). 30 μL of Protein G magnetic beads (Thermo Fisher, 10003D) was added to each reaction, followed by incubation for 2 h at 4°C with rotation. The beads were washed three times with 1 mL of low-salt ChIP wash buffer and once with 1 mL of high-salt ChIP wash buffer for 5 min per wash at 4°C. Chromatin was eluted from the beads with 150 μL of 1 × ChIP Elution Buffer for 30 min at 65°C with gentle shaking. Crosslinks in both immunoprecipitated and input samples were reversed by adding 6 μL of 5 M NaCl and 2 μL of Proteinase K, followed by incubation at 65°C for 2 h. DNA was purified using the spin columns and eluted in 50 μL of DNA Elution Buffer. Purified DNA was analyzed by quantitative PCR using primers spanning the indicated regions of the *IGF2BP1* promoter. All qPCR reactions were performed in technical triplicate. ChIP enrichment was calculated as the percentage of input after correction for the input. HDAC2 occupancy was additionally expressed relative to the corresponding IgG control. Three independent biological experiments were performed.

### Luciferase reporter assay

The *IGF2BP1* reporter plasmid was constructed by inserting the *IGF2BP1* core promoter region into the pGL3-basic vector (Gene Chem). Each group cells were collected and lysed 48 h using passive lysis buffer (Promega, E1941) post-transfection. For each measurement, 10 μL of cell lysate was mixed with 17 μL of firefly luciferase detection reagent, and luminescence was recorded. Subsequently, 17 μL of freshly prepared Renilla luciferase detection working solution was added to the same well to quench the firefly luciferase signal and initiate the Renilla luciferase reaction. Renilla luminescence was recorded and Firefly luciferase activity was normalized to the corresponding Renilla luciferase activity for each well. Relative promoter activity was calculated by setting the normalized value of the indicated control group to 1. Each condition was analyzed in three technical replicate wells, and the experiment was independently repeated three times.

### Methylated RNA immunoprecipitation and quantitative PCR (MeRIP)

Methylated RNA immunoprecipitation followed by quantitative PCR (MeRIP-qPCR) was performed using the Magna MeRIP™ m6A Kit (Millipore, 17−10499) with minor modifications. Total RNA was extracted using TRIzol reagent and treated with DNase I to remove residual genomic DNA. Polyadenylated RNA was isolated from total RNA using VAHTS mRNA Capture Beads (Vazyme, N403-01). All procedures were performed using RNase-free reagents. Purified poly A mRNA was adjusted to approximately 1 μg/μL. Samples were incubated at 94°C for 4 min, and fragmentation was immediately terminated by adding 2 μL of 0.5 M EDTA. Fragmented RNA was placed on ice and purified using an RNA MinElute column. Protein A/G magnetic beads was washed twice and incubated for 30 min at room temperature with either 5 μg of anti-m6A monoclonal antibody (Millipore, MABE1006) or 5 μg of normal mouse IgG (Millipore, CS200621). The antibody-conjugated beads were washed three times. Fragmented RNA was diluted in a total volume of 500 μL containing 5 μL of RNase inhibitor and was then added to the antibody-conjugated beads. The reactions were incubated for 2 h at 4°C with end-over-end rotation. Beads were collected using a magnetic separator and washed three times with 500 μL of ice-cold 1 × IP Buffer. m6A-containing RNA fragments were competitively eluted twice by incubating the beads with 100 μL of m6A-containing Elution Buffer for 1 h at 4°C with continuous mixing. The two eluates were combined, and RNA was purified using an RNeasy MinElute column and eluted in 14 μL of RNase-free water. Quantitative PCR was performed in three independent biological experiments.

### Cell proliferation assay

Cervical cancer cells were cultured in 96-well plates (2000 cells/well) for designated times. According to the manufacturer’s instructions, 10 μL of CCK-8 solution was added to each well and incubated at 37 °C for 3 h. Absorbance at 450 nm was measured.

### 3D spheroid-CD8^+^ T-cell co-culture assay

Cervical cancer cells were transfected with si-IGF2BP1, HDAC2 expression plasmid, or the corresponding negative-control siRNA and empty vector. After 24 h, cells were harvested and seeded into ultra-low attachment round-bottom 96-well plates at 500 cells per well. Tumor spheroids were allowed to form for 48 h at 37°C in a humidified incubator containing 5% CO2. Spheroid formation was monitored by bright-field microscopy, and spheroid size was quantified using ImageJ. For spheroid-CD8^+^ T-cell co-culture, pre-activated human CD8^+^ T cells were added to tumor spheroids at an effector-to-target ratio of 1:1. Pembrolizumab (MCE, MK-3475) or the corresponding IgG control was added at the start of co-culture. The co-culture was maintained for 48 h in complete RPMI-1640 medium. At the endpoint, spheroids were imaged by bright-field microscopy, and spheroid viability was measured using CCK-8 assay according to the manufacturer’s instructions. Culture supernatants were collected and centrifuged to remove debris, and IFNγ concentration was measured by ELISA. Each condition was performed with at least three technical replicates, and the experiment was independently repeated three times.

### Colony formation assay

HeLa and SiHa cells (500 cells/well) were plated in 6-well plates with medium containing 10% FBS and cultured for approximately 2 weeks. Colonies were fixed with 4% paraformaldehyde for 30 min and stained with 0.1% crystal violet for 30 min. Images were captured using a high-definition digital camera.

### Clinical samples

For clinical datasets analysis, studies were considered eligible for our following analysis according to the following criteria: (1) Studies with CC tissue samples. (2) Studies with the presence of normal groups as the control. (3) Studies with patients once received immunotherapy, we selected following datasets from the repository. From the TCGA database (https://tcga-data.nci.nih.gov/tcga/) and the GEO database (https://www.ncbi.nlm.nih.gov/geo/query/acc.cgi?acc=%20GSE253690; https://www.ncbi.nlm.nih.gov/geo/query/acc.cgi?acc=GSE263143;https://www.ncbi.nlm.nih.gov/geo/query/acc.cgi?acc=GSE205247), we downloaded the expression profiles of CC samples. DEGs expression was directly performed between tumor and normal samples with Limma package in R. Similarly, an adjusted P-value cutoff of 1 × 10^−4^ and a fold change cutoff of 1.5 was used to identify target gene. All experiments were conducted in accordance with the Declaration of Helsinki.

### ScRNA‑seq analysis

TISCH (http://tisch.compbio.cn/home/) is a single-cell RNA-sequencing (scRNA-seq) database focusing on the tumor microenvironment (TME) and provides detailed cell-type annotation at the single-cell level. In this research, we used TISCH to analyze the cell subpopulation patterns of immune-response genes from three GEO datasets in CECS. Default options were used for all parameters.

### PMBC-derived CD8 T cells isolation

Peripheral blood mononuclear cells (PBMCs) were purchased from Schbio Biotech (PBMNC100C).

CD8 ⁺ T cells were negatively selected from PBMCs using the Human CD8 ⁺ T Cell Isolation Kit (Miltenyi Biotec, #130-096-495) according to the manufacturer’s instructions, yielding a purity of >95% as determined by flow cytometry. Freshly isolated CD8 ⁺ T cells were activated and expanded for 3 days in RPMI-1640 medium supplemented with 10% fetal bovine serum, 1% penicillin-streptomycin, 100 U/mL recombinant human IL-2 (PeproTech), and pre-coated anti-CD3/CD28 Dynabeads (Gibco, #11161D) at a bead-to-cell ratio of 1:1.

### In vitro co-culture assay

For the co-culture, pre-treated HeLa cells and naive CD8 ⁺ T cells were seeded in 24-well plates at an effector-to-target (E:T) ratio of 5:1 in a final volume of 1 mL of complete RPMI-1640 medium containing 50 U/mL IL-2. To assess the role of PD-1/PD-L1 blockade, soluble anti-PD-1 antibody (Pembrolizumab biosimilar, 10 µg/mL, BioXCell) or an equivalent concentration of human IgG4 isotype control was added to designated wells at the start of co-culture. The co-culture was maintained for 48 hours at 37°C in a 5% CO_2_ incubator and for further viability determination.

### ELISA

The cell culture supernatants were collected and centrifuged to remove debris. The concentrations of secreted human IFN-γ and TNF-α were quantified using specific enzyme-linked immunosorbent assay kits (ELISA, R&D Systems) following the manufacturer’s protocols.

### Statistics and reproducibility

Data were statistically analyzed using GraphPad prism 9.0. Data are presented as mean  ±  standard deviation (SD) from three independent experiments. Comparisons were made using Student’s t-test, one-way analysis of variance (ANOVA) and two-way analysis of variance. Survival differences between groups were compared using Kaplan-Meier (KM) survival analysis. All experiments were repeated at least three times. One-way ANOVA was used to compare the differences among multiple comparisons. A two-way ANOVA analysis was used to calculate the *P* value between groups. Statistical significance was set at P  <  0.05. *P  <  0.05, **P  <  0.01, ***P  <  0.001. NS not significant.

## Result

### Identification of potential immune-response genes for PD-1 therapy in cervical cancer

**Fig 1:**
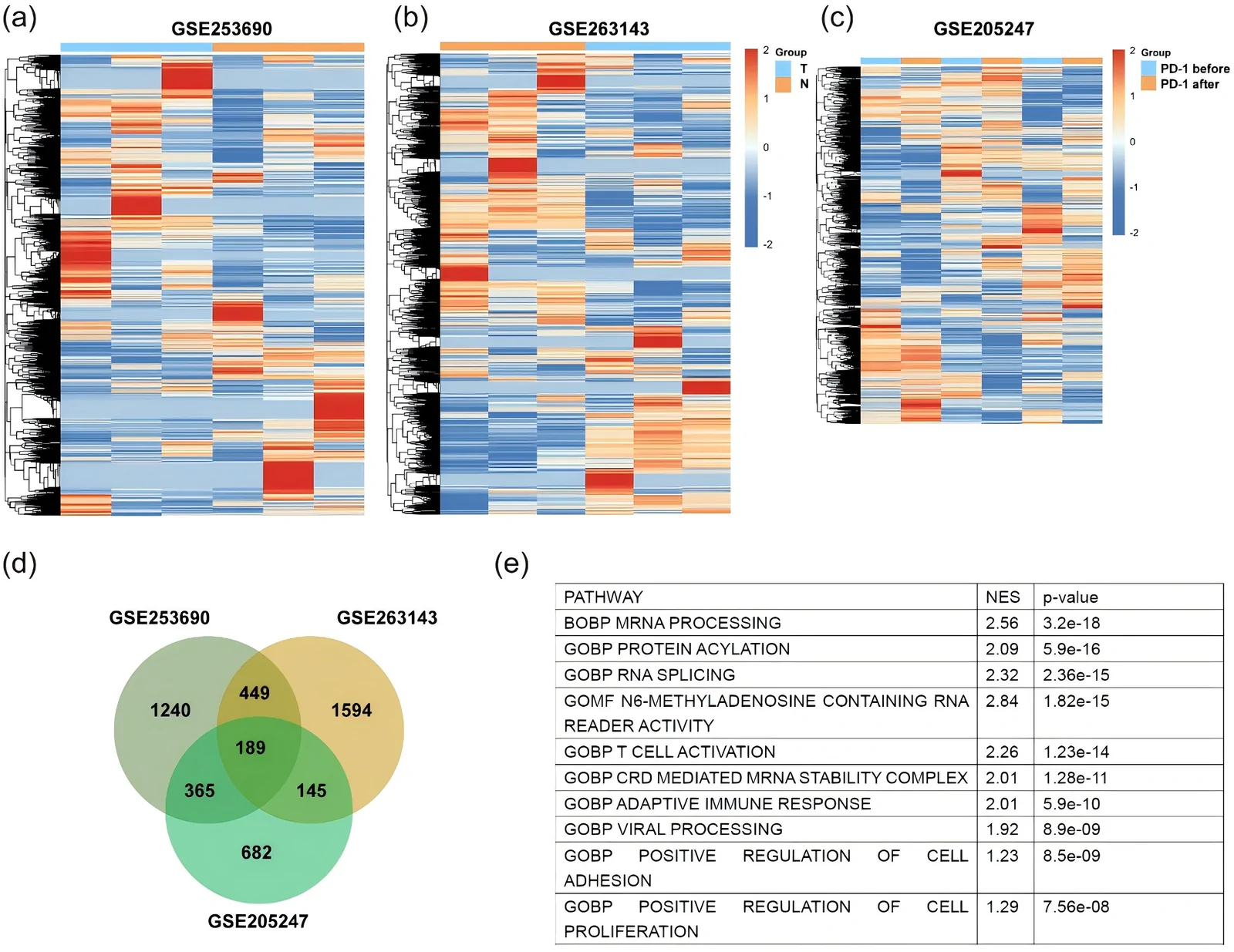
Screening of potential immunotherapy sensitive genes in CC. (a) Heatmap of DEGs of cervical cancer samples in the GSE253690 dataset. (b) Heatmap of DEGs of cervical cancer samples in the GSE263143 dataset. (c) Heatmap of DEGs between samples from patients before or after treated with PD-1 therapy in the GSE205247 dataset. (d) Venn plot depicting overlapped DEGs in GSE253690, GSE263143 and GSE205247. (e) Ranked log2 fold change of genes within the top enriched pathway within GO terms when screening potential PD-1 therapy sensitive targets.

To understand the depth mechanism of PD-1 therapy resistance and explore potential targets in cervical cancer, we downloaded GSE253690, GSE263143 and the GSE205247 which including patients accepted PD-1 treatment. DEGs were extracted with *p* value ≤ 0.01 and common candidates in these three datasets were extracted using Venn plot, and a total of 189 genes were enriched (Fig 1d). Next, we investigated the potential function of enriched genes using the DAVID tool. The results indicated that these genes mainly contribute RNA processing, mRNA m6A modification and adaptive immune response processing (Fig 1e). Together, our results demonstrate mRNA processing participates immunotherapy response in cervical cancer.

### Expression and potential prognostic values of mRNA m6A processing in CC

**Fig 2:**
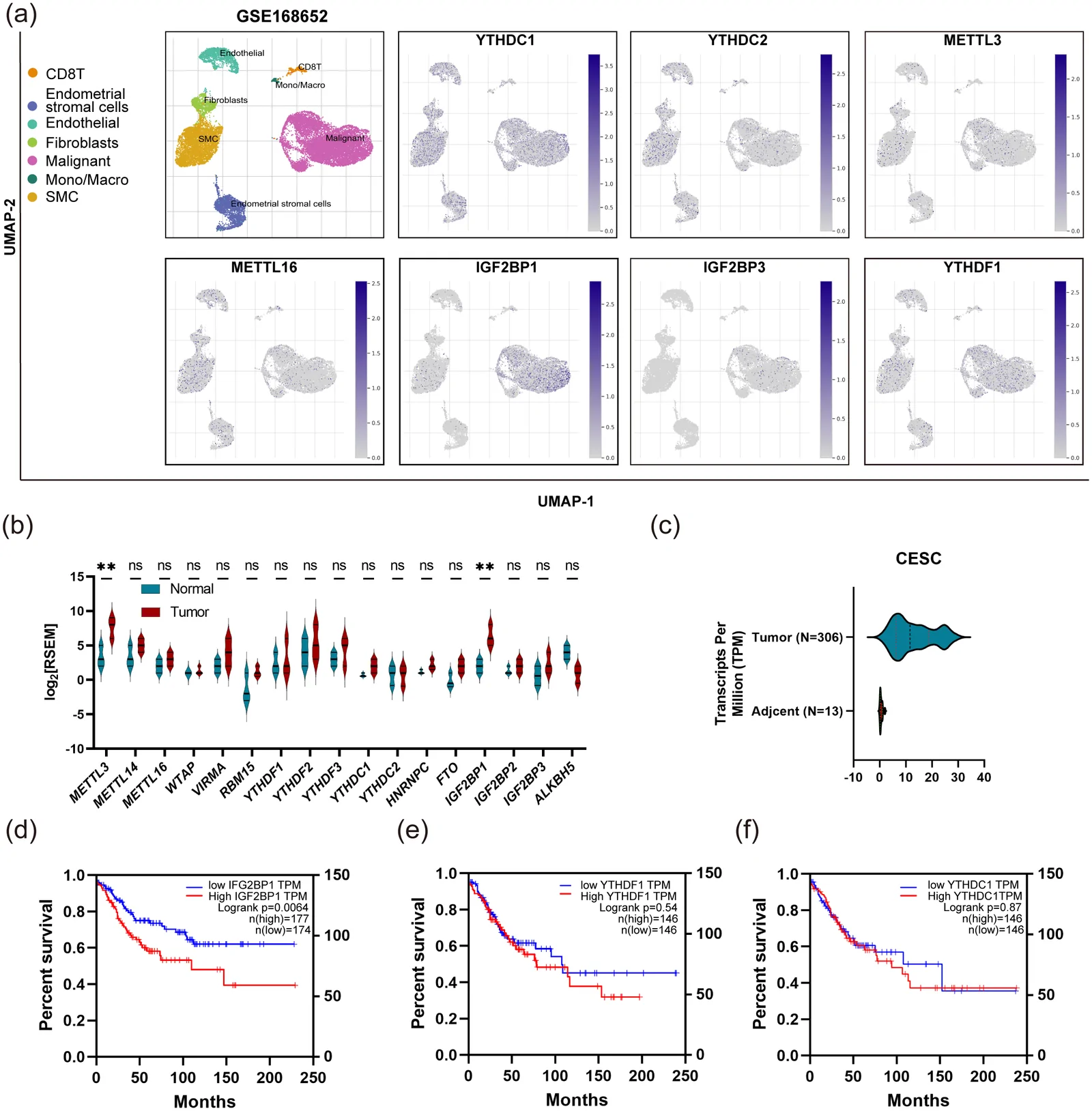
Expression and prognostic values of m6A-related biomarkers. (a) m6A-related genes expression pattern in CC cell subsets. (b) Box-and-whisker plots depicting log2[RSEM] of m6A-related genes from GOMF sets with significant differences between PD-1therapy and Control group patients. (c) Violin plot depicting IGF2BP1 expression in TCGA CESC samples. (d) Overall survival of CC patients expressed different IGF2BP1 by Kaplan-Meier survival analysis in TCGA dataset. (e) Overall survival of CC patients expressed different YTHDF1 by Kaplan-Meier survival analysis in TCGA dataset. (f) Overall survival of CC patients expressed different YTHDC1 by Kaplan-Meier survival analysis in TCGA dataset. **p < 0.01.

To confirm the expression, biological function and potential clinical value of m6A related genes in cervical cancer cells, we firstly investigated the expression pattern of these candidates in malignant cells and microenvironment. Based on the published sc-seq dataset, we found that YTHDC1 was broadly expressed in fibroblasts, immune cells and malignant cells, YTHDF1, YTHDC2, METTL3 and METTL14 were mainly expressed in microenvironment cell contents endothelial and immune cells, only a subset of tumor cells showed positive signal. Interestingly, we noticed that IGF2BP1 exhibits distinct tumor enriched pattern. While IGF2BP3 was lowly expressed in all cell types (Fig 2a). Given the cell number limitation of this dataset, we further analyze the expression of m6A-related genes in TCGA dataset. Most candidates overexpressed in tumor sample and consistent with previous studies. Expectedly, ALKBH5 was low in tumor samples (*n* = 306), indicating specific function in cervical cancer progression. In these genes, IGF2BP1 was most significantly high in tumor samples (Fig 2b and 2c). To evaluate the potential clinical value of aberrant m6A genes in cervical cancer, we utilized GEPIA to obtain survival curve in each cohort. Specifically, IGF2BP1 higher expression cohort patients had worse overall survival (Fig 2d) and no significant survival improvement in YTHDC1 and YTHDF1 low cohort patients (Fig 2e and 2f). These results identify *IGF2BP1* upregulation as a characteristic feature of cervical cancer linked to worse patient survival.

### IGF2BP1 as a pro-tumorigenic factor to promotes proliferation in cervical cancer

**Fig 3:**
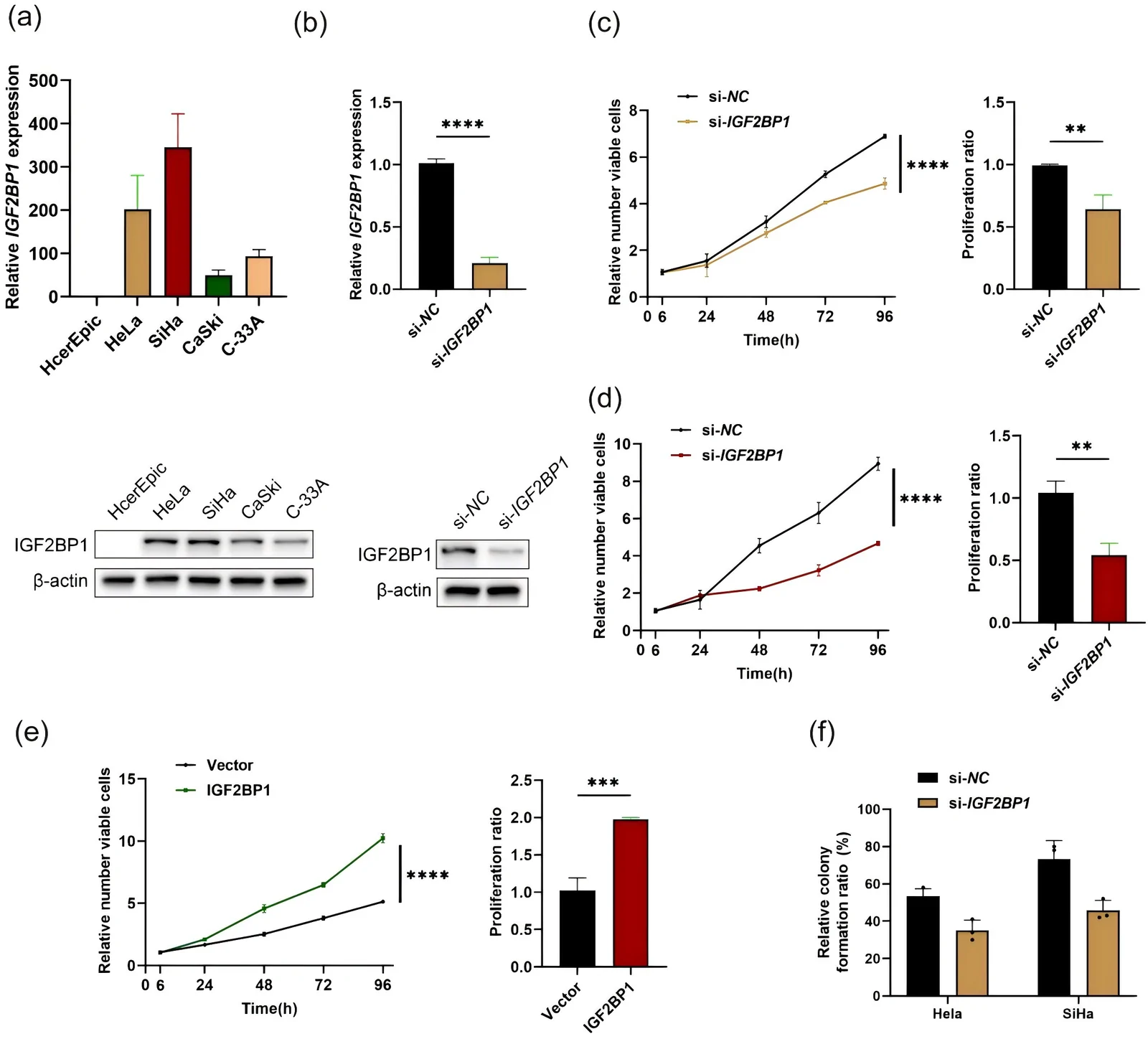
IGF2BP1 promotes the proliferation of cervical cancer cells. (a) The expression of *IGF2BP1* in cervical cancer cell lines and human normal cervical epithelial cell (HcerEpic) was determined by RT-quantitative real-time-polymerase chain reaction (qRT-PCR). (b) The expression of *IGF2BP1* in HeLa cell lines after specific siRNA transfection was determined by RT-quantitative real-time-polymerase chain reaction (qRT-PCR). (c) Cell proliferation assay and determination were conducted in HeLa cells transfected with *IGF2BP1* siRNA or Random control siRNA and following cell counting kit-8 (CCK8) assay. (d) Cell proliferation assay and determination were conducted in HeLa cells transfected with *IGF2BP1* siRNA or Random control siRNA and following cell counting kit-8 (CCK8) assay. (e) Cell proliferation assay and determination were conducted in Hela cells transfected with IGF2BP1 or Vector plasmid and following cell counting kit-8 (CCK8) assay. (f) The effects of *IGF2BP1* siRNA on the proliferation of HeLa and SiHa by colony formation assay. n = 3 independent experiments. **p < 0.01, ***p < 0.001, ****p < 0.0001.

We next used CC cell line as a model to examine the role of IGF2BP1 in cancer cells. IGF2BP1 was aberrantly expressed in HeLa, SiHa, CaSki and C-33A cells, but not normal human cervical epithelial cell line HcerEpic (Fig 3a). Cell viability was evaluated by transfecting HeLa (Fig 3b-c) and SiHa (Fig 3d) cells either the *IGF2BP1* siRNA or random control, followed by cell counting kit-8 (CCK8) and colony formation to analyze cell proliferation. *IGF2BP1* siRNA transfection significantly inhibits cell proliferation (Fig 3b-d and 3f), and colony formation assay demonstrated IGF2BP1 overexpression promotes HeLa cell viability (Fig 3e). Together, these results indicate that IGF2BP1 acts as an oncogene to promote cervical cell proliferation.

### Role of IGF2BP1 on anti-PD-1 blockade

**Fig 4:**
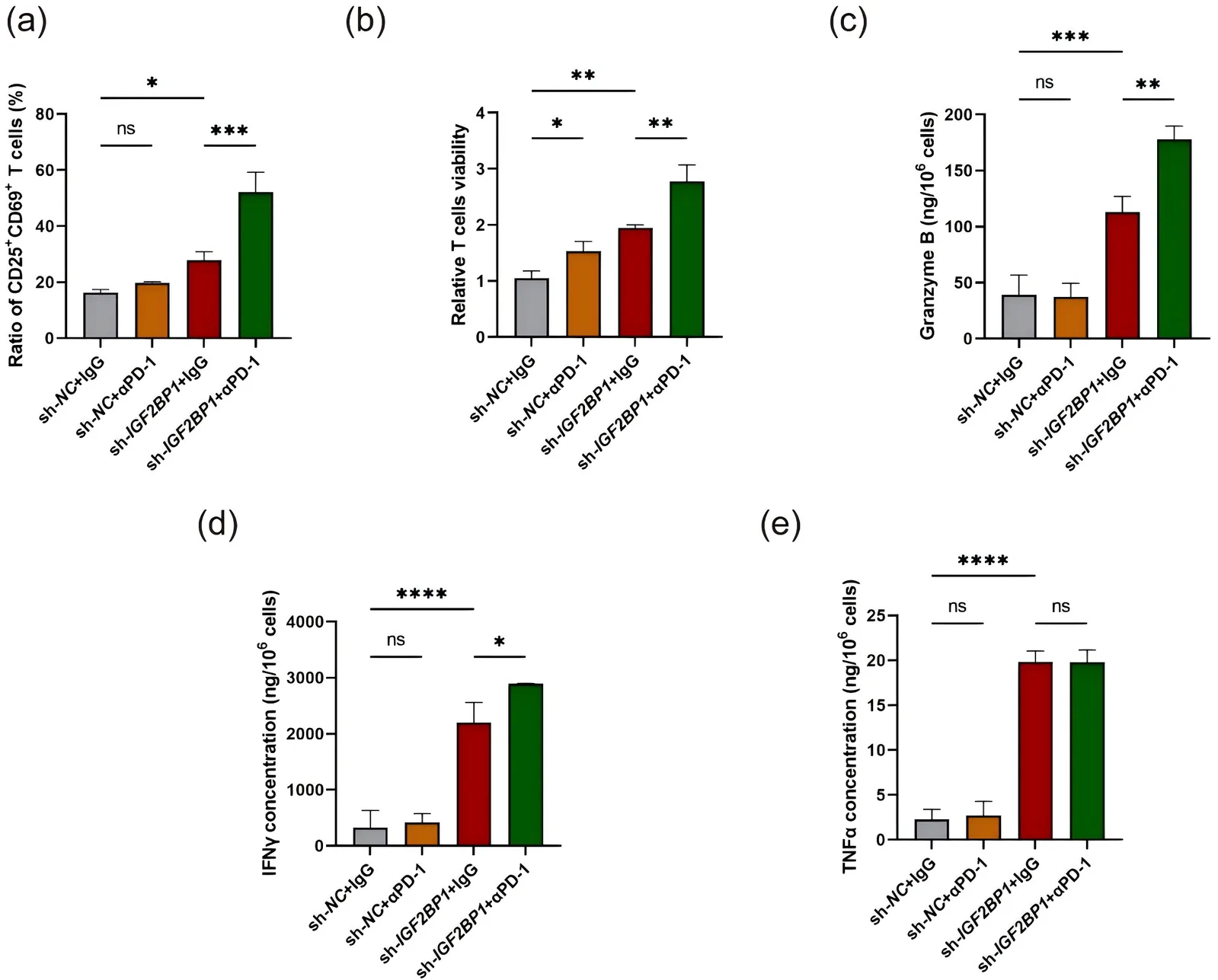
Role of IGF2BP1 in CD8 T cells exhaustion and response to anti-PD-1 antibody in vitro. (a) CD25 and CD69 marked activated CD8^+^ T cells under co-culture with HeLa cells. (b) CCK8 assay to determine CD8^+^ T cells viability under T cells-cancer cells co-culture. (c) Granzyme B expression under co-culture condition. (d) ELISA assay to quantify IFNγ concentration under co-culture and PD-1 antibody incubation. (e) ELISA assay to quantify TNFα concentration under co-culture and PD-1 antibody incubation. n = 3 independent experiments. *p < 0.05, **p < 0.01, ***p < 0.001, ****p < 0.0001.

Although immunotherapy has been effective in producing long-lasting therapeutic effects on several aggressive cancers in subsets of patients, the underline mechanism of immunotherapy resistance still critical to broaden the clinical value for more patients. Given the IGF2BP1 have a distinct role in cervical proliferation and enriched in PD-1 therapy resistant patients, we reasoned that IGF2BP1 may also regulate the response of cervical cancer cells to immunotherapy. To verify whether IGF2BP1 inhibition affects CD8^+^ T cells activation and toxicity function, we established HeLa -T cells co-culture assay. Expectedly, PD-1 antibody incubation showed limited control on CD8^+^ T cells activation in vitro (Fig 4a). In addition, co-cultured with IGF2BP1 knockdown HeLa inhibited CD8^+^ T cells viability (Fig 4b). Cytotoxic T cell recruitment is required for immune response and following tumor cells clearance. To depict the mechanism by which IGF2BP1 silencing sensitizes cervical cancer cells to anti-PD-1 antibody, we proposed that IGF2BP1 knockdown affects T cell function. IGF2BP1 knockdown in HeLa promoted CD8 T cells granzyme B secretion and enhanced PD-1 antibody therapy efficiency (Fig 4c). Subsequent IFNγ and TNFα expression revealed IGF2BP1 is crucial for cancer cells induced T cells activation (Fig 4d). Overall, these data suggest that IGF2BP1 silencing improves CD8 + T cells activation and toxic cytokine release.

### IGF2BP1-dependent m6A methylation regulates aberrant immune-response gene expression in cervical cancer cells

**Fig 5:**
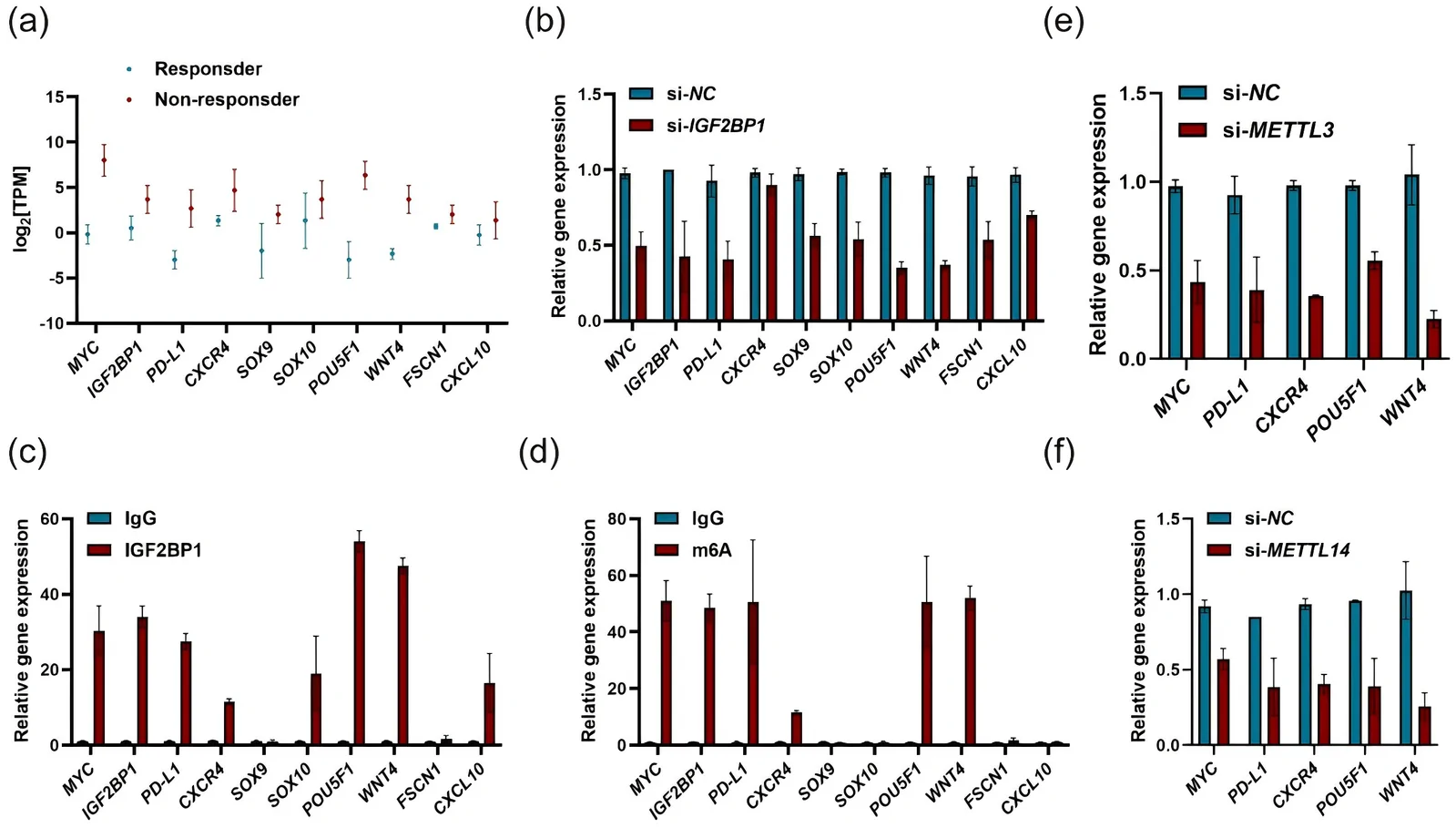
IGF2BP1-dependent m6A methylation regulates aberrant immune-response gene expression in cervical cancer cells. (a) Immune-response genes expression pattern in GSE205247 dataset. (b) qRT-PCR to quantify immune-response genes expression after transfection with *IGF2BP1* siRNA or random control siRNA. (c) RIP-qPCR to detect potential target genes expression binding with IGF2BP1. (d) MeRIP-qPCR to detect m^6^A modification of downstream targets. (e) qRT-PCR to quantify IGF2BP1 binding mRNA expression after transfection with *METTL3* siRNA or random control siRNA. (f) qRT-PCR to quantify IGF2BP1 binding mRNA expression after transfection with *METTL14* siRNA or random control siRNA. n = 3 independent experiments. *p < 0.01, **p < 0.001, ***p < 0.0001.

Next, we wonder the underline mechanism of IGF2BP1 sensitize CC to anti-PD-1 blockade therapy and suppress tumor progression. Previous studies reported that cancer stem cells (CSCs) and stemness program gene upregulation partially contribute to immunotherapy resistance. For validation this proposal, we focused on the stemness program DEGs of GSE205247 including patients performed anti-PD-1 antibody therapy. As expected, *WNT4*, *SOX9* and *POU5F1* (oct4) were significantly enriched in non-response samples (Fig 5a). Additionally, *MYC*, *PD-L1* and pro-inflammatory cytokine *CXCL10* also enriched in PD-1 resistant samples (Fig 5a). Furthermore, we found a significant decrease in most of these genes under IGF2BP1 silencing, indicating direct of indirect regulation of these target genes by IGF2BP1. We performed RNA co-IP and further qRT-PCR demonstrated that the RNA binding protein IGF2BP1 could interact with these target mRNA excluding *SOX9* and *FSCN1* (Fig 5c). As a “reader” of m6A modified mRNA, IGF2BP1 could protect substrate mRNAs from exonucleases and prolong their half-life. Next, we utilized m6A monoclonal antibody to co-IP and conduct qRT-PCR, we identified *WNT4*, *IGF2BP1*, *POU5F1*, *CXCR4*, *PD-L1* and *MYC* proven by recent study, were potential substrates of IGF2BP1 in cervical cancer cells (Fig 5d). Next, we found knockdown of METTL3 and METTL14 decreased theses target genes expression (Fig 5e and 5f). Thus, we revealed that constitutively expressed IGF2BP1 constitutes a critical promoter of key immune-response genes via m6A-dependent approach.

### HDAC2 mediated epigenetic reprogramming sustains constitutive IGF2BP1 activation

**Fig 6:**
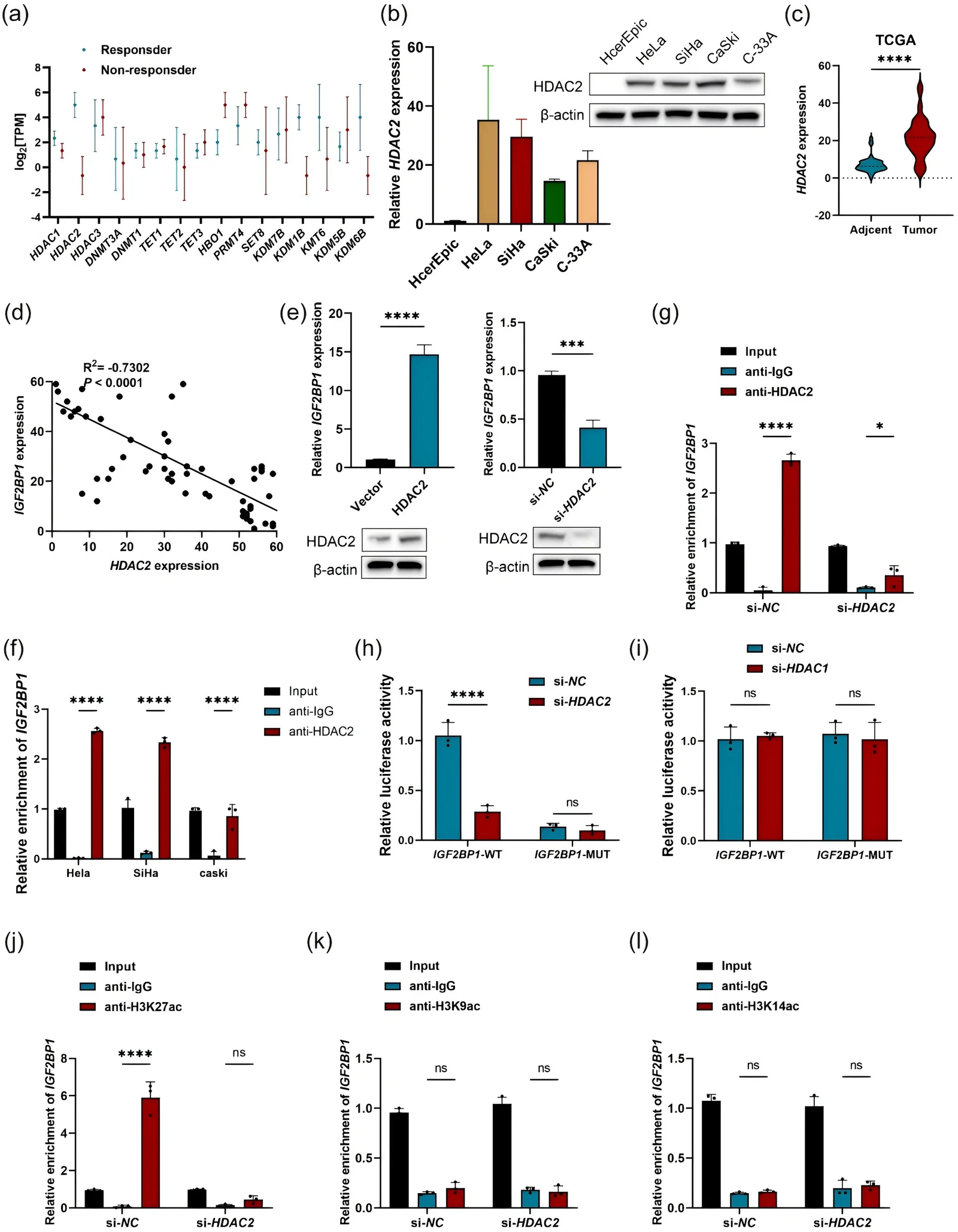
HDAC2 mediated histone deacetylation hampers IGF2BP1 expression in cervical cancer cells. (a) Epigenetic regulator expression in GSE205247 dataset immunotherapy response or non-response patients. (b) The expression of *HDAC2* in cervical cancer cell lines and human normal cervical epithelial cell (HcerEpic) was determined by RT-quantitative real-time-polymerase chain reaction (qRT-PCR) and western blot. (c) Violin plot depicting *HDAC2* expression in TCGA CESC samples. (d) Pearson’s correlation analysis of *HDAC2* and *IGF2BP1* expression in CESC TCGA dataset. (e) qRT-PCR and western blot assays to quantify *IGF2BP1* expression after transfection with *HDAC2* siRNA or 3 × flag-HDAC construct in HeLa cells. (f) CHIP assay was performed to examine the direct interaction of *IGF2BP1* promoter and HDAC2 protein in cervical cancer cells. (g) CHIP-qPCR assay was performed to examine the interaction intensity of *IGF2BP1* promoter with HDAC2 protein in HeLa cells under *HDAC2* knockdown. (h) Dual-luciferase reporter assay to detect the regulation of HDAC2 on IGF2BP1 transcription activation. (i) Dual-luciferase reporter assay to detect the regulation of HDAC1 on IGF2BP1 transcription activation. (j) CHIP-qPCR assay was performed to examine the *IGF2BP1* promoter H3K27ac modification intensity under *HDAC2* knockdown. n = 3 independent experiments. *p < 0.05, ***p < 0.001, ****p < 0.0001.

To elucidate how IGF2BP1 constitutively activated in PD-1 blockade resistant cells, we checked the epigenetic regulator pattern in immunotherapy resistant samples in GEO dataset. Interestingly, HDAC2 is the most enriched genes in response samples (Fig 6a). Previous studies suggested that HDAC2 inhibits suppressor genes transcriptional activation and promotes tumor progression. We found HDAC2 was high expressed in HeLa, SiHa and CaSki cells (Fig 6b). Similarly, HDAC2 expression pattern in TCGA CESC dataset was consistent with previous studies (Fig 6c). However, we identified HDAC2 as an immunotherapy-sensitive gene and may play as a tumor suppressor gene (Fig 6a). These finding represent demonstrate that HDAC2 is highly expressed in tumor tissue, but extremely high expression patients may tend to sensitive to immunotherapy. We wondered whether HDAC2 affects IGF2BP1 expression, person’s correlation analysis revealed significant negative correlation between HDAC2 and IGF2BP1 in advanced CC samples (Fig 6d). Knockdown of HDAC2 increased IGF2BP1 expression in HeLa cells and overexpression of HDAC2 inhibited IGF2BP1 activation in HeLa cells (Fig 6e). ChIP-qPCR detected HDAC2 occupancy at the IGF2BP1 promoter in HeLa, SiHa, and CaSki cells, and this signal decreased after HDAC2 knockdown (Fig 6f and 6g). We assessed the HDAC2 effect on IGF2BP1 transcriptional activation using dual-luciferase reporter system, HDAC2 binds with IGF2BP1 promoter and induces luciferase enzyme activity but not mutant IGF2BP1 promoter construct (Fig 6h). Additionally, HDAC1 could not affect IGF2BP1 promoter activity (Fig 6i). In various HDAC2 catalyzed deacetylation, HDAC2 knockdown caused distinct *IGF2BP1* loci H3K27ac decreasing (Fig 6j). Collectively, these findings imply a context-dependent HDAC2 function in cervical cancer immunotherapy clinical response and HDAC2 inhibits IGF2BP1 expression via H3K27ac.

### Targeting HDAC2/IGF2BP1 axis suppresses PD-1 therapy resistance in CC tumor

**Fig 7:**
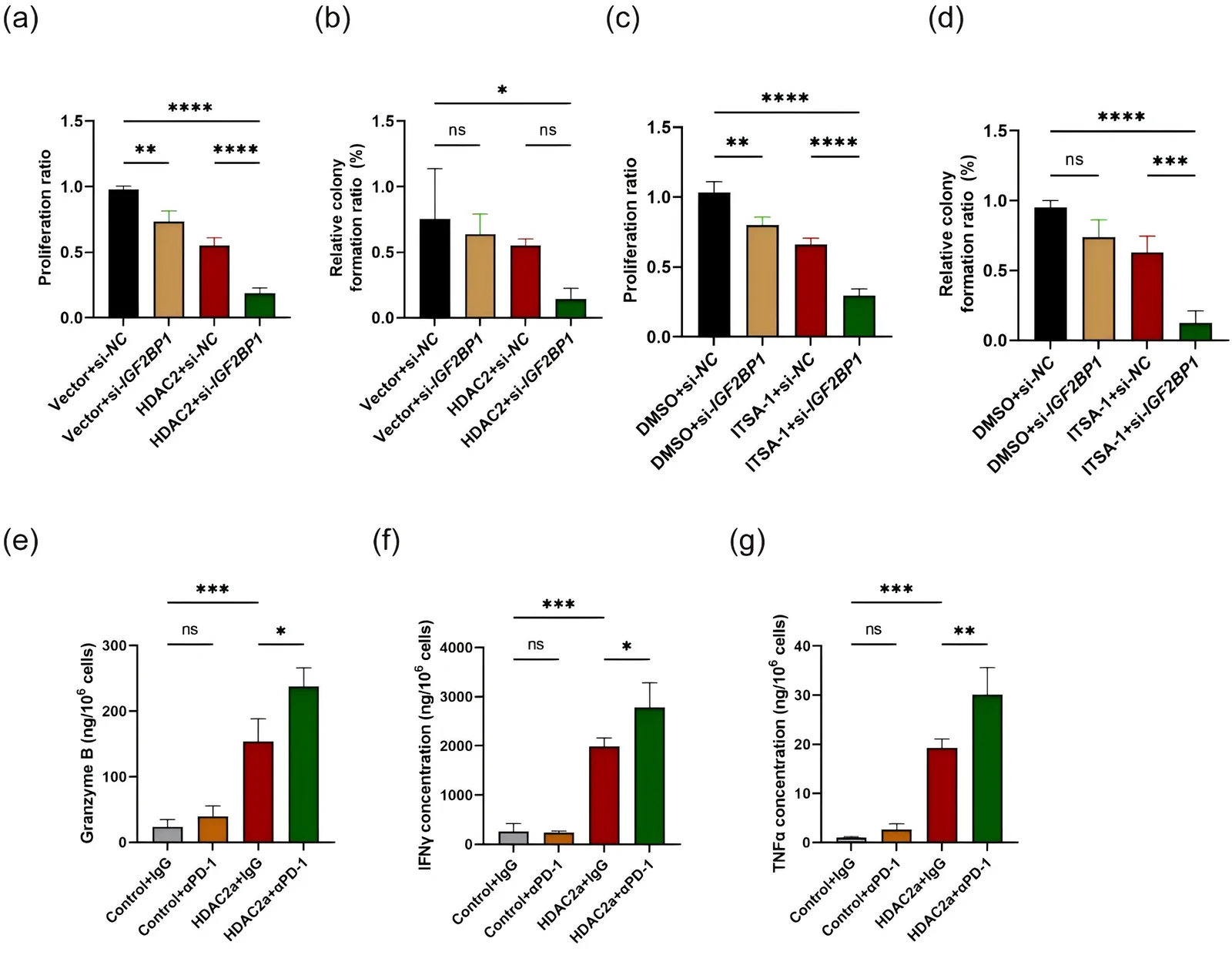
Role of HDAC2/IGF2BP1 in cervical cancer cell response to anti-PD-1 antibody in vitro. (a) Cell proliferation assay was conducted in HeLa cells under HDAC2 overexpression and IGF2BP1 knockdown. (b) The effects of HDAC2 OE and IGF2BP1 KD on the proliferation of HeLa by colony formation assay. (c) Cell proliferation assay was conducted in HeLa cells under IGF2BP1 knockdown and HDAC2 agonist treatment. (d) The effects of combi-therapy on the proliferation of HeLa by colony formation assay. (e) Granzyme B expression of PBMC-derived CD8^+^ T-cells co-cultured with cervical cancer cells. (f) IFNγ expression of PBMC-derived CD8^+^ T-cells co-cultured with cervical cancer cells. (g) TNFα concentration of CD8^+^ T-cells co-cultured with cervical cancer cells. *p < 0.05, **p < 0.01, ***p < 0.001, ****p < 0.0001.

Given the HDAC2/IGF2BP1 axis in CC immunotherapy sensitive program, we then analyzed whether the knockdown of IGF2BP1 and overexpression of HDAC2 hampered tumor cell proliferation and enhanced immune check point blockade therapy efficiency. After HDAC2 overexpression and IGF2BP1 knockdown, we determined the proliferation ratio via CCK8 assay and colony formation assay. As expected, the dual-combi strategy decreased tumor cell proliferation about 90% (Fig 7a-d). To verify whether dual-combi strategy further enhance CD8^+^ T cells activation and anti-cancer function, we utilized in vitro co-culture system. HDAC2 agonist treatment in co-culture system resulted in rising granzyme B, IFNγ and TNFα release (Fig 7d-f). Collectively, these results suggest that surprising HDAC2/IGF2BP1 axis enhance CD8^+^ T cells activation.

### The HDAC2/IGF2BP1 axis modulates anti-PD-1 responsiveness in a 3D spheroid-CD8^+^ T-cell co-culture model

**Fig 8:**
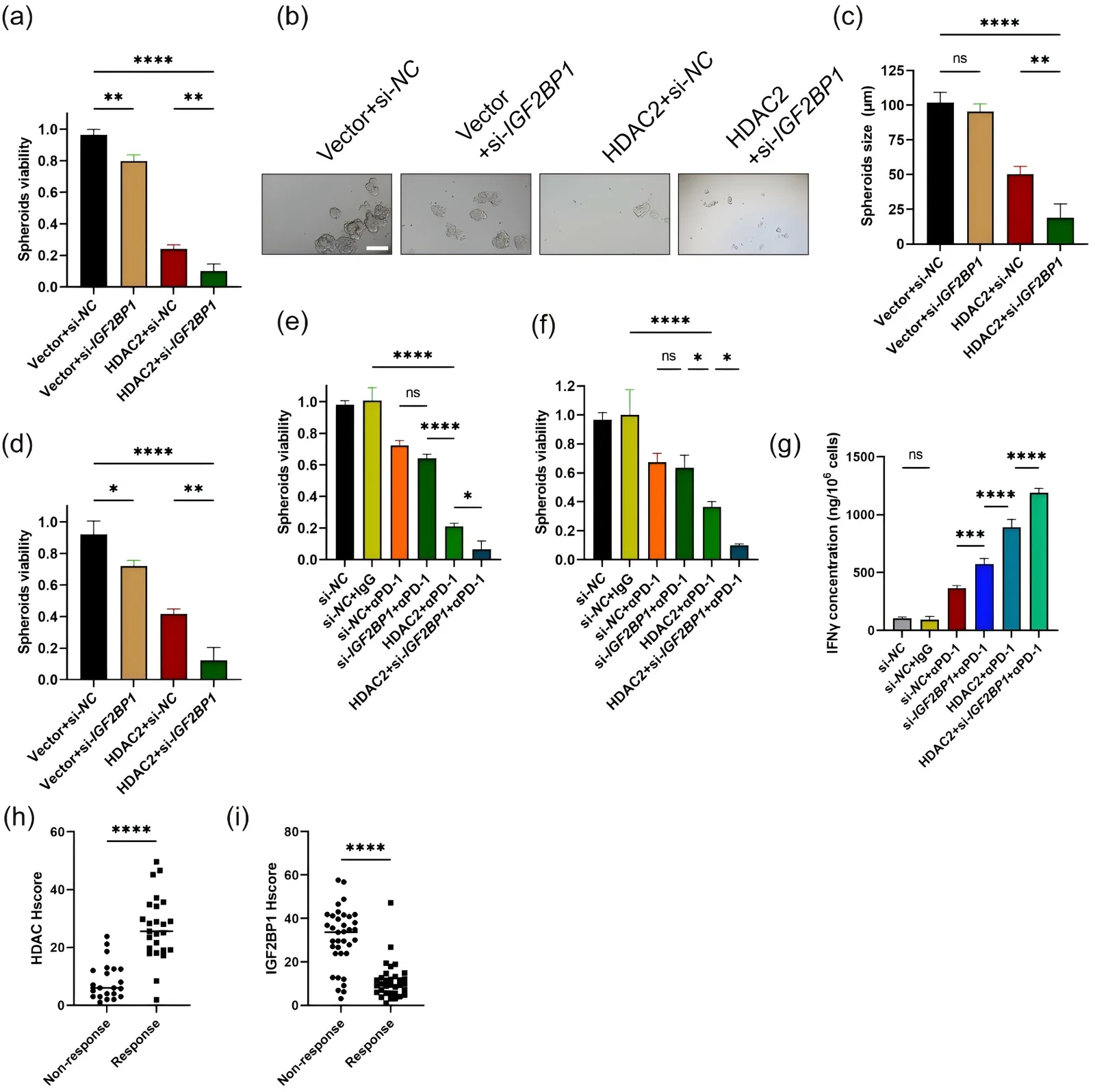
The HDAC2/IGF2BP1 axis modulates CD8 + T-cell-mediated anti-tumor activity in a 3D cervical cancer spheroid co-culture model. (a) Relative viability of HeLa spheroids after transfection with vector control or HDAC2 expression plasmid together with si-NC or si-IGF2BP1under incubated with activated CD8^+^ T cells. (b) Representative bright-field images of co-cultured HeLa spheroids under the indicated conditions. Scale bar, 100 μm. (c) Quantification of co-cultured HeLa spheroid size under the indicated conditions. (d) Quantification of co-cultured C-33A spheroid viability after HDAC2 overexpression and/or IGF2BP1 knockdown. (e, f) Relative viability of HeLa (e) and C-33A (f) spheroids in the spheroid-CD8^+^ T-cell co-culture system after treatment with IgG control or anti-PD-1 antibody. (g) IFNγ concentration in the supernatants of spheroid-CD8^+^ T-cell co-cultures under the indicated treatment conditions. Data are presented as mean ± SD from three independent experiments. (h) HDAC2 expression levels were compared between immunotherapy response or non-response patients in GSE205247. (I) IGF2BP1 expression levels were compared between immunotherapy response or non-response patients in GSE205247. ns, not significant, *P < 0.05, **P < 0.01, ***P < 0.001, ****P < 0.0001.

To further evaluate the functional relevance of the HDAC2/IGF2BP1 axis in a more physiologically relevant in vitro model, we established a 3D cervical cancer spheroid-CD8^+^ T-cell co-culture system. HeLa and C-33A cells were transfected with HDAC2 expression plasmid and/or si-IGF2BP1 and then cultured under low-attachment conditions to generate tumor spheroids, additional activated CD8^+^ T cells were mixed at an optimal of E:T ratio of 1:1. Compared with the vector plus si-*NC* control group, *IGF2BP1* knockdown moderately reduced spheroid viability, whereas HDAC2 overexpression markedly impaired spheroid growth. Combined HDAC2 overexpression and IGF2BP1 knockdown produced the strongest inhibitory effect, as shown by decreased spheroid viability, disrupted spheroid morphology, and reduced spheroid size in HPV positive and negative cells (Fig 8a–d). We next examined whether this regulatory axis affected the response of tumor spheroids to PD-1 blockade. In the spheroid-CD8^+^ T-cell co-culture system, anti-PD-1 treatment alone only moderately reduced spheroid viability. In contrast, *IGF2BP1* knockdown or HDAC2 overexpression further enhanced the inhibitory effect of anti-PD-1 treatment on tumor spheroids. The combination of HDAC2 overexpression and *IGF2BP1* knockdown showed the most pronounced reduction in spheroid viability under anti-PD-1 treatment in two different cell lines (Fig 8e, f). Consistently, IFNγ secretion in the co-culture supernatant was increased after *IGF2BP1* knockdown or HDAC2 overexpression and was highest in the combined HDAC2 overexpression plus IGF2BP1 knockdown group treated with anti-PD-1 antibody (Fig 8g). These results support that the HDAC2/IGF2BP1 axis may influences CD8^+^ T-cell-associated anti-tumor activity and anti-PD-1 responsiveness in a 3D cervical cancer spheroid model.

## Discussion

Despite advances in PD-1/PD-L1 based immunotherapy success in subsets of tumor patients, CC patients still had no improvement [22,23]. Here we found that the m6A reader, IGF2BP1 plays an important role in CC. IGF2BP1 is increased in CC, suggesting a pro-tumorigenic role of IGF2BP1 in CC. IGF2BP1 promotes proliferation in cervical cancer cells in vitro and tumor growth in vivo. Our mechanism study reveals that IGF2BP1 increases m6A enrichment in the immune response and stemness-promoting mRNA including *PD-L1, WNT4*, *CXC4*, *IGF2BP1* and *POU5F1*, and increases their mRNA stability. The regulation of these genes by IGF2BP1 is mediated through m6A demethylation validated through *METTL3* and *METTL14* knockdown. In addition, IGF2BP1 plays important role in immunotherapy resistance via downstream genes expression regulation such as PD-L1 and following immune cells recruitment and T cells activation. Furthermore, IGF2BP1 can be downregulated by HDAC2 mediated H3K27ac erasure. Our study identifies HDAC2 as a context-dependent regulator in cancer progression and enhance CD8 T cells activation in CC immunotherapy.

IGF2BP1, which belongs to the IGF2BP RNA-binding protein family that including IGF2BP1, IGF2BP2 and IGF2BP3, has been shown to regulate various oncogenic processes via stabilization of the substrate m6A modified mRNA, such as *MYC* in our data [24,25]. Recently, Shi and his colleague identified *BTBD7* and *HEG1* as novel IGF2BP1 targets and defined RPTM3/IGF2BP1/HEG1 axis as important regulator in oxaliplatin resistance [26]. Study in NSCLC found m6A modified *circNDUFB2* function as IGF2BP1 substrate and further enhance TRIM25 mediated IGF2BP1 destabilization [27]. While IGF2BP1 is established as an oncogenic m6A “reader” that stabilizes *MYC* and *SOX2* mRNAs in endometrial cancer, its role in shaping tumor-immune crosstalk remains poorly defined. Our data demonstrate N6-methyladenosine modified stemness program genes also are substrates of IGFBP1 by which increasing gene expression and may promote immune evasion via enhanced cancer stem cells [28]. Additionally, our data may suggest IGF2BP1 function as oncogene in various tumor types via context-dependent substrate mRNA.

In past decade, numerous studies found interaction of programmed cell death 1ligand 1 (PD-L1) and corresponding receptor programmed cell death 1 (PD-1) could inhibit T cell response [29–32]. Further, such interaction has proven abolish immunotherapy efficiency [27]. Also, recent studies have reported different forms of PD-L1, such as soluble protein in exosome could inhibit antitumor immune response [28]. In our study, we found IGF2BP1 promotes PD-L1 expression in a m6A-dependent manner, further supports the potential of IGF2BP1 as a biomarker of immunotherapy resistance.

HDAC1 and HDAC2 are two highly homologous Class I HDACs and display specific roles in different cell types or response to various stimulation [28]. Recent progress has highlighted the importance of HDAC2 in tumor initiation and advanced progression [33,34]. Numerous studies have shown that HDAC2 expression was upregulation in tumor samples compared with its matched normal tissues [34–36]. Recent finding reported HDAC2 knockout compromises IFNγ-induced H3K27, H3K9 deposition and BRD4 recruitment in PD-L1 promoter, further suppress immune escape [36]. Consistently, we identified that HDAC2 was upregulated in CC samples. In addition, our multi-omics analysis revealed HDAC2 as the top-ranked epigenetic sensitizer in immunotherapy-responsive CC, indicating HDAC2 potential marker of immunotherapy sensitivity in cervical cancer patients. Mechanistic studies confirming its direct repression of IGF2BP1 transcription via H3K27 deacetylation. Crucially, we may redefine HDAC2 as a context-dependent tumor suppressor. Similarly, recently study found that HDAC1 and HDAC2, two core members of HDAC family, play tumor-suppressive function in T cells during anaplastic large cell lymphoma (ALCL) development [37]. La Noce and colleagues found that HDAC2 depletion promotes osteosarcoma stemness and CSC phenotypes both in vitro and in vivo [19]. Our study focused on the epigenetic modulation axis instead of HDAC2-direct downstream genes and found HDAC2-mediated histone modification directly affect mRNA modification and its novel function in cervical cancer immunotherapy response. All these studies position HDAC2 as a context-dependent regulator in cancer progression and relapse.

However, there were some limitations in this study. First, while TCGA/GEO analyses support clinical relevance, prospective validation of association of HDAC2/IGF2BP1 axis with immunotherapy response in larger, uniformly treated cervical cancer cohorts is essential. Second, the impact of high-risk HPV subtypes (e.g., HPV16 vs HPV18) on the HDAC2/IGF2BP1 axis needs investigation, given their potential influence on tumor immunogenicity. Third, the lack of mouse model validation, we alone test CD8^+^ T cells activation in vitro. In future study, we will establish immunocompetent cervical cancer models (e.g., HPV-transgenic mice) or IGF2BP1-KO CC model to strength our tri-combi-therapy. Forth, we will further investigate the tumor or cell type specificity of HDAC2 suppressor function in immune-response.

## Conclusion

In summary, we have uncovered a previously unrecognized HDAC2/IGF2BP1 axis and downstream immune response genes expression cascade that critically governs cervical cancer progression and anti-PD-1 immunotherapy resistance. HDAC2 functions as a transcriptional brake on IGF2BP1, dampening downstream gene expression, PD-L1 decreasing, and CD8^+^ T cell recruitment, thereby fostering a positive immune tumor microenvironment. Different with previous study, HDAC2 could as a biomarker of immunotherapy response, higher HDAC2 implies positive anti-PD-1 immunotherapy response. In addition, our study positions IGF2BP1 as potential actionable targets for further address its clinical availability.

## Supporting information

S1 File siRNA and shRNA sequences, qPCR primer sequences, and information on publicly available datasets used in this study. (DOCX)

S2 File Original Western blot images. Original and uncropped Western blot images corresponding to Figs 3a, 3b, 6b, and 6e.
(DOCX)
