# Supplementary material for: HDAC2 restrains IGF2BP1-associated m6A dysregulation in cervical cancer immunotherapy resistance
Source: PLoS One. 2026 Aug 27;21(8):e0355606. doi: 10.1371/journal.pone.0355606 (PMC13521371; doi:10.1371/journal.pone.0355606)
Supplement: Supplementary file 1 [file pone.0355606.s001.docx]

**siRNA sequence used in this study:**

IGF2BP1 siRNA: 5’-CAUCAGAAGGGACAGAGUA-3’; 5’- GACAACAACGGGCAGAAAU-3’; 5’- UGAGGUGGUAGUACCAAGA-3’

HDAC2 siRNA: 5’- CAGGAGACUUGAGGGAUAU-3’; 5’- GAGAUGGUAUAGAUGAUGA-3’; 5’- CAAAGGUGAUGGAGAUGUA-3’

METTL3 siRNA: 5’- GCAAGUAUGUUCACUAUGA-3’; 5’- GCAAGAAUUCUGUGACUAU-3’; 5’- GCUCAACAUACCCGUACUA-3’

METTL14 siRNA：5’- GGAUGAGUUAAUAGCUAAA-3’; 5’- CCUAUUUCAGUGCUCCUAA-3’; 5’- GGACUUGGGAUGAUAUUAU-3’

**shRNA sequence used in this study:**

IGF2BP1 shRNA 1#: 5’- CTCCAAAGTTCGTATGGTTAT’;

IGF2BP1 shRNA 2#: 5’- GATGGATGCTACGAGTATAAA’.

**Primer used in this study**

| Gene name | Forward primer | Reverse primer |
| --- | --- | --- |
| MYC | GCCACGTCTCCACACATCAG | TGGTGCATTTTCGGTTGTTG |
| IGF2BP1 | GATTGCACCACCCGAAACAC | GGGTCTCCAGCTTCACTTCC |
| PD-L1 | TGGCATTTGCTGAACGCATTT | TGCAGCCAGGTCTAATTGTTTT |
| CXCR4 | CAGCAGGTAGCAAAGTGACG | GTAGATGGTGGGCAGGAAGA |
| SOX9 | AGCGAACGCACATCAAGAC | CTGTAGGCGATCTGTTGGGG |
| SOX10 | AGGCTGCTGAACGAAAGTGA | CAAGTGGGCGCTCTTGTAGT |
| POU5F1 | TCCCTTCGCAAGCCCTCAT | CACCACCTGGAGGGGGCG |
| WNT4 | ACCTGGAAGTCATGGACTCG | TCAGAGCATCCTGACCACTG |
| FSCN1 | CAGCGGCCTCTCGTCTAC | GTACTTGTTGCCGCAGTTGA |
| CXCL10 | CTCCAGTCTCAGCACCATGA | GCTCCCCTCTGGTTTTAAGG |

**Publicly available data**

The dataset used in this study includes TCGA database (https://tcga-data.nci.nih.gov/tcga/) and the GEO database (https://www.ncbi.nlm.nih.gov/geo/query/acc.cgi?acc=%20GSE253690; https://www.ncbi.nlm.nih.gov/geo/query/acc.cgi?acc=GSE263143; https://www.ncbi.nlm.nih.gov/geo/query/acc.cgi?acc=GSE205247).

we used TISCH (http://tisch.compbio.cn/home/) to analyze the cell subpopulation patterns of immune-response genes from three GEO datasets in CECS.
