# Supplementary material for: HDAC2 restrains IGF2BP1-associated m6A dysregulation in cervical cancer immunotherapy resistance
Source: PLoS One. 2026 Aug 27;21(8):e0355606. doi: 10.1371/journal.pone.0355606 (PMC13521371; doi:10.1371/journal.pone.0355606)
Supplement: Supplementary file 2 [file pone.0355606.s002.docx]

**Figure 3a&b (actin)**

Actin 42kDa

HcerEpic; HeLa; SiHa; CaSki; C-33A

**Extended Data Fig.1** **The original blotting of actin in Fig.3a. Left, original blotting of actin and corresponding sample names; Right, cropped version in manuscript.**

Actin 42kDa

si-*NC*; si-*IGF2BP1*

**Extended Data Fig.2 The original blotting of actin in Fig.3b. Left, original blotting of actin and corresponding sample names; Right, cropped version in manuscript.**

IGF2BP1 63kDa

HcerEpic; HeLa; SiHa; CaSki; C-33A

**Extended Data Fig.3 The original blotting of IGF2BP1 in Fig.3a. Left, original blotting of actin and corresponding sample names; Right, cropped version in manuscript.**

si-*NC*; si-*IGF2BP1*

IGF2BP1 63kDa

**Extended Data Fig.4 The original blotting of IGF2BP1 in Fig.3b. Left, original blotting of actin and corresponding sample names; Right, cropped version in manuscript.**

**Figure 6b&e**

HcerEpic; HeLa; SiHa; CaSki; C-33A

Actin 42kDa

**Extended Data Fig.5 The original blotting of actin in Fig.6b. Left, original blotting of actin and corresponding sample names; Right, cropped version in manuscript.**

Vector; HDAC2

Actin 42kDa

**Extended Data Fig.6 The original blotting of actin in Fig.6e Left panel. Left, original blotting of actin and corresponding sample names; Right, cropped version in manuscript.**

si-*NC*; si-*HDAC2*

Actin 42kDa

**Extended Data Fig.7 The original blotting of actin in Fig.6e Right panel. Left, original blotting of actin and corresponding sample names; Right, cropped version in manuscript.**

HcerEpic; HeLa; SiHa; CaSki; C-33A

HDAC2 60kDa

**Extended Data Fig.8 The original blotting of HDAC2 in Fig.6b. Left, original blotting of actin and corresponding sample names; Right, cropped version in manuscript.**

HDAC2 60kDa

Vector; HDAC2

**Extended Data Fig.9 The original blotting of HDAC2 in Fig.6e Left panel. Left, original blotting of actin and corresponding sample names; Right, cropped version in manuscript.**

si-*NC*; si-*HDAC2*

HDAC2 60kDa

**Extended Data Fig.10 The original blotting of HDAC2 in Fig.6e Right panel. Left, original blotting of actin and corresponding sample names; Right, cropped version in manuscript.**
